# Supplementary material for: Functional and analytical recapitulation of osteoclast biology on demineralized bone paper
Source: Nat Commun. 2023 Dec 7;14:8092. doi: 10.1038/s41467-023-44000-9 (PMC10703810; doi:10.1038/s41467-023-44000-9)
Supplement: Supplementary file 1 — Supplementary Information [file 41467_2023_44000_MOESM1_ESM.pdf]

Supplementary Information

Functional and analytical recapitulation of osteoclast biology on demineralized bone paper

Yongkuk Park[1], Tadatoshi Sato[2], Jungwoo Lee[1,3,4]

[1]Department of Chemical Engineering, Institute for Applied Life Sciences, University of Massachusetts, Amherst, MA 01003, USA.
[2]Department of Medicine, UMass Chan Medical School, Worcester, MA 01605, USA
[3]Department of Biomedical Engineering, University of Massachusetts, Amherst, MA 01003, USA
[4]Molecular & Cellular Biology Graduate Program, University of Massachusetts, Amherst, MA 01003, USA

Corresponding author: Jungwoo Lee, jungwoo@umass.edu

Supplementary Figure

Supplementary Figure 1. Comparative OB gene expression profiles between TCP and DBP.
Supplementary Figure 2. TRAP staining of fixed OCs after 6-day of VD3/PGE2 stimulation.
Supplementary Figure 3. Quantified OC number after 7-day of RANKL/M-CSF stimulation.
Supplementary Figure 4. Characterization of osteoclastogenesis on bone particle-coated plate.
Supplementary Figure 5. Quantitative imaging analysis algorithm of identifying OCs.

Supplementary Data

Supplementary Data 1. Osteoblasts RNA sequencing results paired (i) TCP vs. DBP and (ii) resting vs. VD3/PGE2 stimulation.

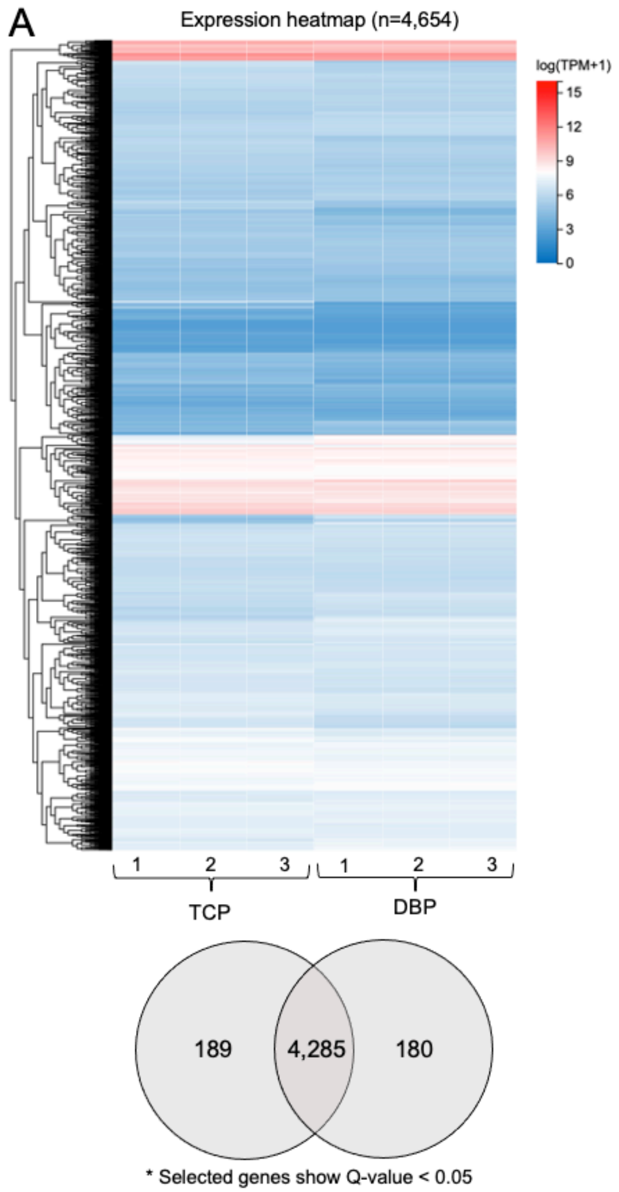

B

Top 30 upregulated genes		
LOC118568284	LOC118568644	Heyl
Pramel13	Fcrla	Trpc3
Serpina1a	Cnmd	Kera
Cryaa	Ngfr	Nkx3-2
D130043K22Rik	Spock3	Ly6d
Gm6348	Gm36718	Npy1r
Ces3a	Padi4	Mup14
Apoa1	Csmd1	Ptger3
Klk1b11	Gadl1	Cd4
Apba2	Grm7	Serpina3k
**Top 30 downregulated genes**		
Gm4963	Wnt7b	Col13a1
Ptx4	Krt18	Ccdc194
Lrrc55	Prn	Bmp8a
Gm45978	Pcdha9	4933428M09Rik
Cyp26a1	Slc6a15	Scube1
Tecta	Gpr37l1	Col26a1
Fgf23	Grrp1	Slc9a2
Dmp1	Fxyd6	Ptprb
Megf10	Hhip	Gli1
Gm15682	Rpl17-ps8	Lhfpl1

Supplementary Figure 1. Comparative osteoblast gene expression profiles between TCP and DBP. (A) RNA-sequencing heatmap comparing gene expression profiles of osteoblasts between TCP and DBP for selected genes with the Q-value lower than 0.01 (total: 4,654 genes). Distinct and overlapped gene numbers between TCP and DBP. **(B)** The list of top 30 up- and down-regulated genes between TCP and DBP culture. (up-regulated: Log2fc > 1, down-regulated: Log2fc < -1).

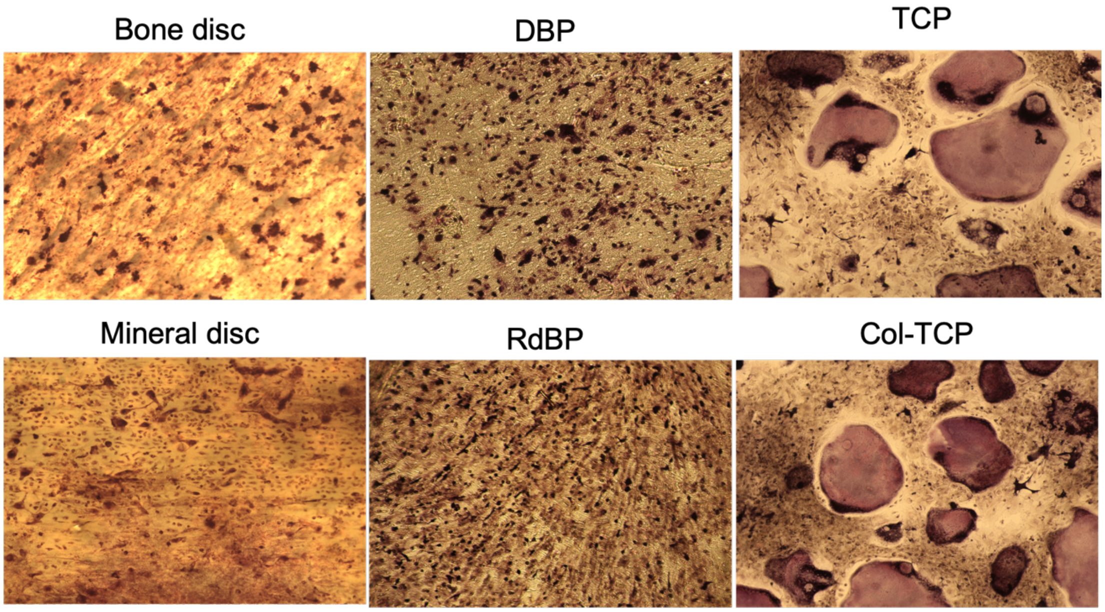

Supplementary Figure 2. Tartrate-resistant acid phosphatase (TRAP) staining of fixed osteoclast cultures on different substrates after 6 days of VD3/PGE2 stimulation.

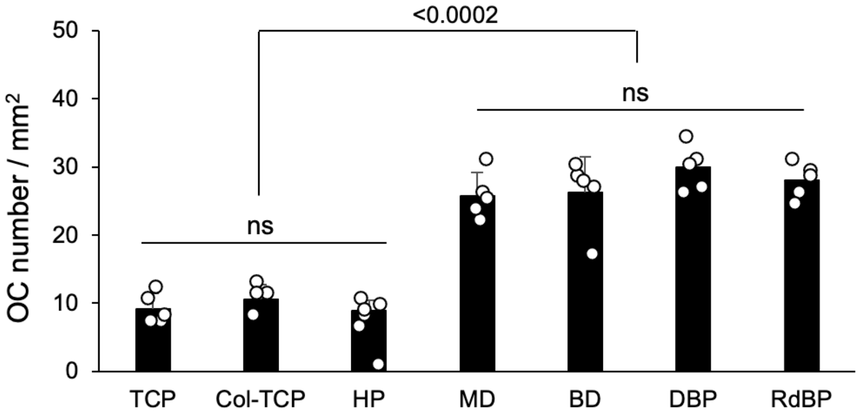

Supplementary Figure 3. Quantified osteoclast number after 7 days of RANKL/M-CSF stimulated culture on different substrates. The inner panel shows osteoclast size after 14 days of stimulated culture on RdBP (n=5 independent samples). Source data are provided as a Source Data file.

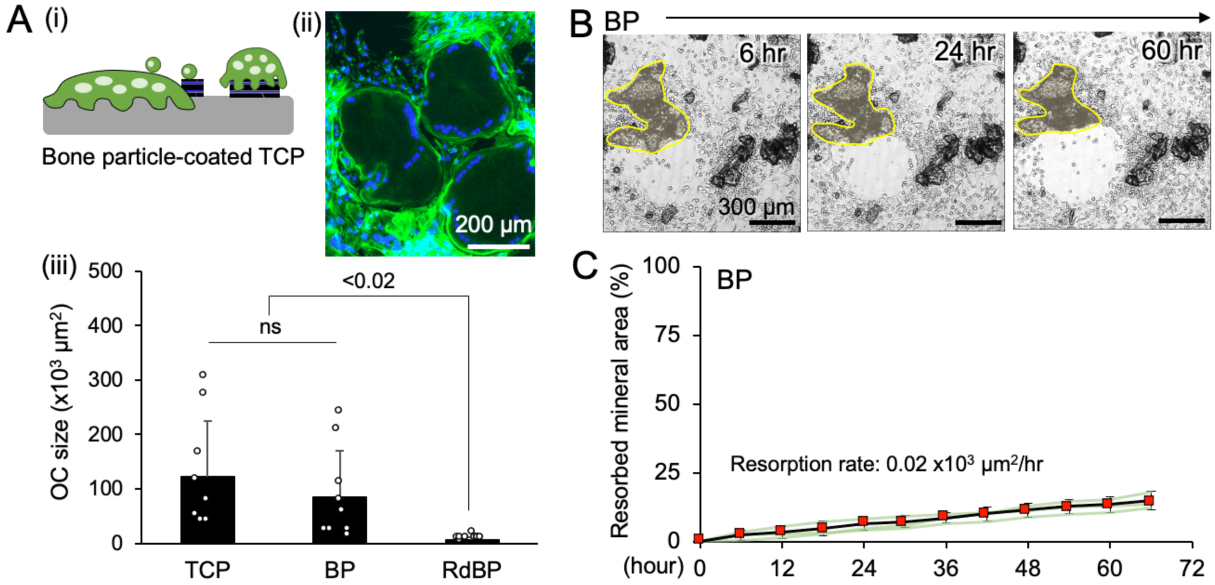

Supplementary Figure 4. Characterization of osteoclastogenesis on bone particle-coated plate (BP). (i) Schematic and (ii) actin-stained images of osteoclasts on BP, and (iii) comparison of osteoclast size on BP, TCP, and RdBP. Data are shown as mean ± SD. P-values from unpaired two-tailed t-tests are indicated. ns: not significant (p >0.05). (n=9 representative data from 3 independent experiments). (iv) Time-lapse microscopic monitoring of osteoclastic mineral resorption on BP. (v) Time-course measurements of osteoclastic mineral resorption area on BP with a bone resorption rate (n=5 independent samples). Source data are provided as a Source Data file.

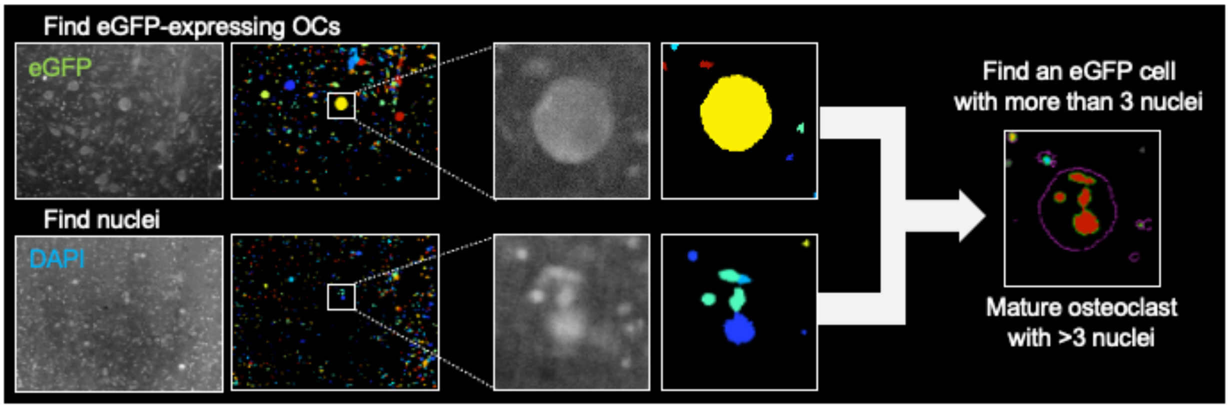

Supplementary Figure 5. Quantitative imaging analysis algorithm of identifying OCs. eGFP+ large BMMs were identified, and cell nuclei were stained with DAPI. Individual channel images were processed to optimize the intensity and contrast by using CellProfiler. Then, fluorescent signals were identified as objects. An eGFP+ cell including more than 3 nuclei was counted as a mature osteoclast.
