# Supplementary material for: Functional and analytical recapitulation of osteoclast biology on demineralized bone paper
Source: Nat Commun. 2023 Dec 7;14:8092. doi: 10.1038/s41467-023-44000-9 (PMC10703810; doi:10.1038/s41467-023-44000-9)
Supplement: Supplementary file 3 — Description of Additional Supplementary Files [file 41467_2023_44000_MOESM3_ESM.pdf]

Description of Additional Supplementary Files

File Name: Supplementary Data 1
Description: Osteoblasts RNA sequencing results paired (i) TCP vs. DBP and (ii) resting vs. VD3/PGE2 stimulation.

File Name: Supplementary Movie 1
Description: Z-stack images of mineral deposition on DBP by murine OBs. Deposited mineral was stained with Calcein, while the actin structure of mouse osteoblasts was labeled with Alexa Fluor 568. Mineralized DBP was observed using second harmonic generation imaging of a multiphoton microscope.

File Name: Supplementary Movie 2
Description: Time-laps images of OC mineral resorption on RdBP. Resorption trajectories of osteoclasts were observed on Calcein-stained RdBP, distinguishing pit- (left) and trench-(right) type patterns by the decreased fluorescent intensity of Calcein.

File Name: Supplementary Movie 3
Description: Time-laps images of OC differentiation and mineral resorption on TCP, HP, BP, and RdBP. No resorption occurred on TCP due to the absence of mineral. Osteoclastic resorption of a hydroxyapatite layer on TCP (HP) and human bone particles on TCP (BP) were observed under an optical microscope. Osteoclastic resorption of calcein-stained RdBP was observed under fluorescent microscope.

File Name: Supplementary Movie 4
Description: Time-lapse images of OB-OC coculture on TCP and DBP under VD3/PGE2 stimulation. DsRed osteoblasts and eGFP MNCs were cocultured on TCP (left) and DBP (Right) with VD3/PGE2 stimulation.

File Name: Supplementary Movie 5
Description: Time-lapse images of OB-OC coculture on TCP and DBP withdrawal VD3/PGE2. DsRed osteoblasts and eGFP osteoclasts were cocultured for 6 days with VD3/PGE2 stimulation, and then VD3/PGE2 were withdrawn. Without VD3/PGE2 stimulation, eGFP osteoclasts on TCP (left) and DBP (right) gradually underwent fission and apoptosis.

File Name: Supplementary Movie 6
Description: Time-lapse images of OC fusion during co-culture with OBs on DBP. eGFP osteoclast precursors fused during OB-OC coculture with VD3/PGE2 stimulation.

File Name: Supplementary Movie 7
Description: Time-lapse images of OC fission during co-culture with OBs on DBP. Cell nuclei were stained with a live cell nucleus (DAPI) staining reagent. Multinucleated osteoclasts divided into multiple cells during fission after VD3/PGE2 withdrawal.

File Name: Supplementary Movie 8
Description: Time-lapse images of OC mineral resorption on single culture on RdBP and coculture with OBs on DBP. Calcein-stained RdBP demonstrated mineral resorption with decreased fluorescent intensity during BMM single culture on RdBP in the presence of M-CSF and RANKL (left) and OB-OC coculture on DBP under VD3/PGE2 stimulation (right).

File Name: Supplementary Movie 9
Description: Time-lapse images of OB-OC co-culture on DBP without and with OS680 treatment. DsRed OBs and eGFP OCs were cocultured with (right) and without (left) OS680 treatment. Apoptotic osteoclasts exhibited a rapid shrinkage of cell cytoplasm, eventually disappearing from the surface.

File Name: Supplementary Movie 10
Description: Time-lapse images of caspase-3/7 apoptosis of OB-OC co-culture on DBP without and with OS680 treatment. DsRed OCs were cocultured with B6 OBs on DBP with (right) and without (left) OS680 treatment in the presence of green caspase-3/7 dye.
