# Supplementary material for: Functional and analytical recapitulation of osteoclast biology on demineralized bone paper
Source: Nat Commun. 2023 Dec 7;14:8092. doi: 10.1038/s41467-023-44000-9 (PMC10703810; doi:10.1038/s41467-023-44000-9)
Supplement: Supplementary file 15 — Reporting Summary [file 41467_2023_44000_MOESM15_ESM.pdf]

nature portfolio

Corresponding author(s): Jungwoo Lee

Last updated by author(s): Nov 20, 2023

Reporting Summary

Nature Portfolio wishes to improve the reproducibility of the work that we publish. This form provides structure for consistency and transparency in reporting. For further information on Nature Portfolio policies, see our Editorial Policies and the Editorial Policy Checklist.

Statistics

For all statistical analyses, confirm that the following items are present in the figure legend, table legend, main text, or Methods section.

n/a	Confirmed	
☐	☒	The exact sample size (*n*) for each experimental group/condition, given as a discrete number and unit of measurement
☐	☒	A statement on whether measurements were taken from distinct samples or whether the same sample was measured repeatedly
☐	☒	The statistical test(s) used AND whether they are one- or two-sided<br>*Only common tests should be described solely by name; describe more complex techniques in the Methods section.*
☐	☒	A description of all covariates tested
☐	☒	A description of any assumptions or corrections, such as tests of normality and adjustment for multiple comparisons
☐	☒	A full description of the statistical parameters including central tendency (e.g. means) or other basic estimates (e.g. regression coefficient) AND variation (e.g. standard deviation) or associated estimates of uncertainty (e.g. confidence intervals)
☐	☒	For null hypothesis testing, the test statistic (e.g. *F*, *t*, *r*) with confidence intervals, effect sizes, degrees of freedom and *P* value noted<br>*Give P values as exact values whenever suitable.*
☒	☐	For Bayesian analysis, information on the choice of priors and Markov chain Monte Carlo settings
☒	☐	For hierarchical and complex designs, identification of the appropriate level for tests and full reporting of outcomes
☒	☐	Estimates of effect sizes (e.g. Cohen's *d*, Pearson's *r*), indicating how they were calculated

Our web collection on statistics for biologists contains articles on many of the points above.

Software and code

Policy information about availability of computer code

Data collection	Microscope imaging: ZEN imaging software (ZEISS), NIS-Elements (Nikon), Lumaview720 (Etaluma), M7000 (EVOS)<br>MicroCT imaging: Living Animal (Perkin Elmer), Fiji (Image J)<br>Mechanical stiffness: XEI software (TA Instruments)<br>Microplate reader: Gen 5 (BioTek)
Data analysis	Quantitative data: Microsoft Office Excel (2016)<br>Imaging data: MATLAB_r2020a, Fiji (Image J), EVOS analysis (EVOS), CellProfiler (Broad Institute, 2021)<br>RNA-seq analysis: SOAPnuke (BGI), Spliced Alignment of Transcripts (HISAT) software, DESeq2, Dr.Tom system (BGI).

For manuscripts utilizing custom algorithms or software that are central to the research but not yet described in published literature, software must be made available to editors and reviewers. We strongly encourage code deposition in a community repository (e.g. GitHub). See the Nature Portfolio guidelines for submitting code & software for further information.

Data

Policy information about availability of data

All manuscripts must include a data availability statement. This statement should provide the following information, where applicable:

- Accession codes, unique identifiers, or web links for publicly available datasets
- A description of any restrictions on data availability
- For clinical datasets or third party data, please ensure that the statement adheres to our policy

The RNA sequencing data generated in this study have been deposited in the NCBI Sequence Read Archive under accession code PRJNA104146. Source data are provided as a Source Data file. Supplementary Data is provided RNA seq results. Supplementary Movies (1-10) and Supplementary Figures (1-5) are provided.

Research involving human participants, their data, or biological material

Policy information about studies with human participants or human data. See also policy information about sex, gender (identity/presentation), and sexual orientation and race, ethnicity and racism.

Reporting on sex and gender	De-identified healthy human male and female donors fresh bone marrow aspirates and peripheral blood are obtained from commercial vendors to isolate primary human bone marrow stromal cells and CD14+ monocytes.
Reporting on race, ethnicity, or other socially relevant groupings	n/a
Population characteristics	n/a
Recruitment	n/a
Ethics oversight	n/a

Note that full information on the approval of the study protocol must also be provided in the manuscript.

Field-specific reporting

Please select the one below that is the best fit for your research. If you are not sure, read the appropriate sections before making your selection.

☒ Life sciences ☐ Behavioural & social sciences ☐ Ecological, evolutionary & environmental sciences

For a reference copy of the document with all sections, see nature.com/documents/nr-reporting-summary-flat.pdf

Life sciences study design

All studies must disclose on these points even when the disclosure is negative.

Sample size	As a standard practice for the field,statistical analysis were performed on data derived from at least 3 biological replicates.<br>The detailed sample size for each experiment is shown in all the figure legends.
Data exclusions	No data were excluded.
Replication	All experiments were individually repeated 2-3 times in order to confirm reproducibility.
Randomization	Animals and samples were chosen randomly for each experimental batch.<br>Cells were cultured and maintained in the same environment and randomly allocated to each group.
Blinding	MicroCT imaging was conducted by an individual who was blinded to the specific groups of control and treated samples in the experiment.<br>In other experiments, the investigator was not blinded to the experimental groups, as it was necessary to differentiate between the control and treatment groups.

Reporting for specific materials, systems and methods

We require information from authors about some types of materials, experimental systems and methods used in many studies. Here, indicate whether each material, system or method listed is relevant to your study. If you are not sure if a list item applies to your research, read the appropriate section before selecting a response.

Materials & experimental systems

n/a	Involved in the study
☐	☒ Antibodies
☒	☐ Eukaryotic cell lines
☒	☐ Palaeontology and archaeology
☐	☒ Animals and other organisms
☒	☐ Clinical data
☒	☐ Dual use research of concern
☒	☐ Plants

Methods

n/a	Involved in the study
☒	☐ ChIP-seq
☒	☐ Flow cytometry
☒	☐ MRI-based neuroimaging

Antibodies

Antibodies used

Anti-OPG (Mouse) antibody was used for ELISA (DY459, R&D systems), Anti-RANKL (Mouse) antibody was used for ELISA (DY462, R&D systems), Anti-CD14 (Human) antibody was used for CD14 positive cell selection (8802-6834, Invitrogen).
We followed the vendors guideline to dilute these capture antibodies for ELISA and cell sorting.

Validation

All antibodies were validated by the manufactures. We used only purchased antibodies that were prevalidated by these companies.

Animals and other research organisms

Policy information about studies involving animals; ARRIVE guidelines recommended for reporting animal research, and Sex and Gender in Research

Laboratory animals

DsRed mice were obtained from B. Osborne (UMass-Amherst). C57BL/6 (000664) and GFP mice (003291) were obtained from the Jackson Laboratory. Male and female mice aged between 4 to 8 weeks were randomly selected for each bath of the experiment. Mice were maintained at the UMass-Amherst core animal facility with controlled environmental settings, including a 12-hour light/12-hour dark cycle. Ambient temperatures were kept at 22°C, and relative humidity was regulated at 50 ± 10%. Continuous monitoring was performed to ensure a stable and suitable environment for the mice's welfare, managed by the core facility's trained animal care staff.

Wild animals

This study did not use wild animals.

Reporting on sex

Both male and female mice were used.

Field-collected samples

This study did not involve field-collected samples.

Ethics oversight

All animal procedures were approved by the Institutional Animal Care and Use Committee of the University of Massachusetts Amherst.

Note that full information on the approval of the study protocol must also be provided in the manuscript.

Plants

Seed stocks

Report on the source of all seed stocks or other plant material used. If applicable, state the seed stock centre and catalogue number. If plant specimens were collected from the field, describe the collection location, date and sampling procedures.

Novel plant genotypes

Describe the methods by which all novel plant genotypes were produced. This includes those generated by transgenic approaches, gene editing, chemical/radiation-based mutagenesis and hybridization. For transgenic lines, describe the transformation method, the number of independent lines analyzed and the generation upon which experiments were performed. For gene-edited lines, describe the editor used, the endogenous sequence targeted for editing, the targeting guide RNA sequence (if applicable) and how the editor was applied.

Authentication

Describe any authentication procedures for each seed stock used or novel genotype generated. Describe any experiments used to assess the effect of a mutation and, where applicable, how potential secondary effects (e.g. second site T-DNA insertions, mosiacism, off-target gene editing) were examined.
